# Correction: Synthesis of nanostructured catalysts by surfactant-templating of large-pore zeolites

**DOI:** 10.1039/c9na90019e

**Published:** 2019-03-28

**Authors:** Aqeel Al-Ani, Josiah J. C. Haslam, Natalie E. Mordvinova, Oleg I. Lebedev, Aurélie Vicente, Christian Fernandez, Vladimir Zholobenko

**Affiliations:** School of Chemical and Physical Sciences, Keele University Keele Staffordshire ST5 5BG UK a.a.t.al-ani@keele.ac.uk v.l.zholobenko@keele.ac.uk; Oil Marketing Company (SOMO) Baghdad Iraq; Laboratoire CRISMAT, ENSICAEN, UMR CNRS 6508 6 Boulevard du Maréchal Juin 14050 Caen Cedex 04 France; Normandie Univ, ENSICAEN, UNICAEN, CNRS, Laboratoire Catalyse et Spectrochimie 14000 Caen France

## Abstract

Correction for ‘Synthesis of nanostructured catalysts by surfactant-templating of large-pore zeolites’ by Aqeel Al-Ani *et al.*, *Nanoscale Adv.*, 2019, DOI: 10.1039/c9na00004f.

The authors wish to make the following correction to the manuscript:

The units of the molar absorption coefficients quoted in the experimental section should be “cm micromol^−1^” (or cm μmol^−1^) instead of “cm mol^−1^”.

The Royal Society of Chemistry apologises for these errors and any consequent inconvenience to authors and readers.

## Supplementary Material

